# Outcome and prognostic factors in 593 non-metastatic rectal cancer patients: a mono-institutional survey

**DOI:** 10.1038/s41598-018-29040-2

**Published:** 2018-07-16

**Authors:** Julien Langrand-Escure, Peng Diao, Max-Adrien Garcia, Guoping Wang, Jean-Baptiste Guy, Sophie Espenel, Elodie Guillaume, Amel Rehailia-Blanchard, Grégoire Pigné, Guy de Laroche, David Kaczmarek, Thierry Muron, Jack Porcheron, Jean-Marc Phelip, Alexis Vallard, Nicolas Magné

**Affiliations:** 1 Department of Radiation Oncology, Lucien Neuwirth Cancer Institute, 108 bis, Avenue Albert Raimond, BP 60008, 42271 Saint-Priest en Jarez, France; 2 Department of Radiation Oncology, Sichuan Cancer Hospital, Chengdu, 610041 Sichuan Province China; 3 Public Health Department, Hygée Institute, Avenue Albert Raimond, BP 60008, 42271 Saint-Priest en Jarez, France; 4 Thoracic and Digestive Surgery Department, Private Loire Hospital (HPL), 39 Boulevard de la Palle, 42100 Saint-Étienne, France; 5 Department of Medical Oncology, Lucien Neuwirth Cancer Institute, 108 bis, Avenue Albert Raimond, BP 60008, 42271 Saint-Priest en Jarez, France; 6 Department of Digestive and Oncologic Surgery, North University Hospital, Avenue Albert Raimond, 42271 Saint-Priest en Jarez, France; 7 Hepatology and Gastroenterology Department, North University Hospital, Avenue Albert Raimond, 42271 Saint-Priest en Jarez, France

## Abstract

This retrospective study was undertaken to provide more modern data of real-life management of non-metastatic rectal cancer, to compare therapeutic strategies, and to identify prognostic factors of overall survival (OS) in a large cohort of patients. Data on efficacy and on acute/late toxicity were retrospectively collected. Patients were diagnosed a non-metastatic rectal cancer between 2004 and 2015, and were treated at least with radiotherapy. OS was correlated with patient, tumor and treatment characteristics with univariate and multivariate analyses. Data of 593 consecutive non-metastatic rectal cancer patients were analyzed. Median follow-up was 41 months. Median OS was 9 years. Radiotherapy was delivered in pre-operative (n = 477, 80.5%), post-operative (n = 75, 12.6%) or exclusive (n = 41, 6.9%) setting. In the whole set of patients, age, nutritional condition, tumor stage, tumor differentiation, and surgery independently influenced OS. For patients experiencing surgery, OS was influenced by age, tumor differentiation and nodal status. Surgical resection is the cornerstone treatment for locally-advanced rectal cancer. Poor tumor differentiation and node involvement were identified as major predictive factor of poor OS. The research in treatment intensification and in identification of radioresistance biomarkers should therefore probably be focused on this particular subset of patients.

## Introduction

The current standard of care for locally advanced rectal cancer (RC) is a pre-operative chemo-radiation, followed by a total mesorectal excision (TME). Radiotherapy historically improved RC local control, with a superiority of pre-operative *versus* post-operative radiotherapy on local recurrence rate^1–3^. As for surgery, TME decreased the local recurrence risk from 30% to 10–15% and improved survival rate^4–6^. However most of the overall survival (OS) prognosis factors were identified with post-operative radiotherapy programs and before TME was systematically performed. This is a major limitation for prognosis factors analysis since publications suggested that the RC modern outcome was much better than it used to be, when trials assessing the benefits of adjuvant therapy were recruiting^7^. The development of staging, surgery, radiotherapy, pathological examination and multidisciplinary teams might have significantly improved the outcome of non-metastatic RC patients. Thus, it is of paramount importance to identify current prognosis factors in real-life patients with non-metastatic RC, since such factors play a part in deciding on the optimal treatment plan, and might play a future crucial role in achieving personalized anticancer treatments and follow-up. Moreover, since the debate between a “long” chemoradiotherapy and a “short” exclusive accelerated radiotherapy (25 Gy/5fr) is not yet concluded, the analysis of real-life prescription behaviours in a French university health centre might bring interesting information.

The aim of the present study was to describe the outcome and the management of care in one of the largest cohort of non-metastatic RC patients. The identification of prognosis factors of overall survival (OS) was retrospectively performed in the whole set of patients, and in the subset of patients undergoing rectal tumor resection.

## Methods and Materials

A retrospective study was conducted at the Lucien Neuwirth comprehensive cancer care center (Saint Priest en Jarez, France). The institutional review board approved the study, which was conducted in accordance with the Helsinki Declaration. Informed consent stating that patient’s medical data would be used to conduct retrospective studies was systematically obtained before radiation initiation.

### Patient population

Medical records of all consecutive patients undergoing radiotherapy for a non-metastatic RC between 2004 and 2015 were retrospectively reviewed. Patient characteristics (age, sex, ECOG performance status, body mass index (BMI)), tumor histology and staging, radiotherapy characteristics (treated locations, dose, fractionation, pre- or post-operative setting), administered chemotherapy, resulting acute and late toxicities, surgery characteristics (complete sterilization of the operative specimen (ypCR), complete tumor resection (R0)) were also studied.

### Treatment definition

#### Chemo-radiotherapy association

Chemo-radiation was defined as concurrent when chemotherapy was overlapping radiotherapy. Chemo-radiation was defined as sequential when chemotherapy was not overlapping the pre- or post-operative radiotherapy.

#### Radiation therapy

Patients were treated in supine position, and immobilized using leg-positioning foamed wedges. CT-scan images were acquired without contrast agent infusion with a slice thickness of 2.5 mm. Plans were contoured and calculated using the Eclipse treatment planning system (Varian Medical Systems, Palto Alto, CA). Gross tumor volume (GTV), clinical tumor volume (CTV), planning tumor volume (PTV) and organs at risk (OAR) were delineated based on planning-CT. Their definition evolved with the availability and development of CT-scan and MRI, and with the delineation guidelines’ editions. 3D conventional radiotherapy was generally used, and intensity modulated radiotherapy (IMRT) was exceptionally performed, in case of special dose constraints (previous history of radiotherapy…). In each case, treatment plans were optimized according to dose limits for OAR and constraints for volume coverage i.e. PTV should receive 95% to 107% of the prescribed dose. Rectal equivalent 2 Gy (EQD2) dose was calculated using the EQD2 formula provided by Fowler^8^ and α/β = 6.2^9^.

#### Surgery

Surgery was defined by a tumor resection. Defunctioning stoma was not considered as surgery.

### Evaluation of efficacy, acute and late toxicities

Follow-up was calculated from the completion of radiotherapy. Patients were assessed for toxicity every week during RT course, and every 3 months later. Radiation-related toxicities were retrospectively graded using the Common Terminology Criteria for Adverse Events v4.0 (CTCAEv4.0)^10^. For each patient, only the most severe toxicity was collected. Acute toxicity was defined by a toxicity occurrence within 3 months from the beginning of radiotherapy. Late toxicity occurred after 3 months, and could also be reported by surgeons and/or general practitioners. Chemotherapy-induced toxicities were collected in medical oncology files. After radiotherapy completion, patients were assessed for efficacy every 3 months by surgeons and medical oncologists during the first two years and every 6 months later, with clinical examination and alternation of chest/abdomen/pelvis- CT-scan and chest radiography and abdominal ultrasound.

### Statistical analysis

Progression free survival (PFS) was defined as the time from the date of radiotherapy completion to the date of clinical and/or radiological RC progression. Overall survival (OS) was defined as the time from the date of radiotherapy completion to the date of death or the last follow-up. Patients and patient’s oncologists were systematically contacted in 2015 to update the patient’s status. PFS and OS were estimated with the Kaplan-Meier method. Survivals were then compared based on log-rank test. Median values were given with the first and third quartiles (Q1-Q3) or with the range. Chi-2 test or Fisher test were performed to compare patient characteristics distribution. All *p* values were nominal without adjustment for multiple testing. Significance was defined by *p* < 0.05. The multivariate analysis was performed using a Cox multivariate analysis based on the significant -or close-to-significance (*p* < 0.2)- factors in univariate analysis. Associated factors (*i.e*. Chi-2 tests: p < 0.001) were excluded. The multivariate model was refined using the Akaike information criterion. Statistical analyses were processed with R 3.2.2 (R Core Team (2013, The R Foundation for Statistical Computing, Vienna, Austria).

### Data availability

The datasets generated during and/or analysed during the current study are available from the corresponding author on reasonable request. Patient indivudal data is provided in Supplementary file.

## Results

### Patient characteristics

Data of 593 consecutive metastasis-free patients undergoing radiotherapy between 2004 and 2015 for a proven RC were analyzed. At time of radiotherapy, median age was 68.8 years (range: 27–96), with 362 males and 231 females. Patients were in good condition, with 549 patients (92.6%) with an ECOG Performance Status 0–1. Malnutrition (BMI < 18.5) was reported in 21 patients (3.5%). Most of RCs (n = 507, 85.5%) were diagnosed at a locally advanced setting (stage II-III). Tumors were located in the lower (n = 252, 42.5%), middle (n = 249, 42%) or upper (88, 14.8%) rectum. The most frequent histology was adenocarcinoma (n = 587, 99%), with moderate differentiation (n = 278, 46.9%). A rectal surgery was performed in 552 patients (93.1%). Patient and tumor characteristics are reported in Table 1.

**Table 1:** Patient and tumor characteristics.

Number of patients	Whole set of patients n (%)
	593 (100)
Median age, years (range)	68.8 (27–96)
≤70 years, n (%)	316 (53.3)
>70 years, n(%)	277 (46.7)
Gender, n (%)	
Male	362 (61)
Female	231 (39)
Sex ratio: Male/Female	1.5
WHO Performance Status, n (%)	
0–1	549 (92.6)
2–3	44 (7.4)
Body mass index, n (%)	
<18.5	21 (3.5)
18.5–29.9	502 (84.7)
≥30	70 (11.8)
Location, n (%)	
Lower rectum	252 (42.5)
Middle rectum	249 (42.0)
Upper rectum	88 (14.8)
Missing data	4 (0.7)
Tumor Histology, n (%)	
Adenocarcinoma	587 (99.0)
Other	6 (1.0)
Tumor differenciation, n (%)	
Well	229 (38.6)
Moderate	278 (46.9)
Poor	31 (5.2)
Missing Data	55 (9.3)
Radiologic tumor staging, n (%)	
Stage I	49 (8.2)
Stage II	192 (32.4)
Stage III	315 (53.2)
Missing data (Tx and/or Nx and M0)	37 (6.2)
Primary tumor resection, n (%)	
Yes	552 (93.1)
No	41 (6.9)

Q1: first quartile, Q3: third quartile, n: number of patients, Tx: unknown tumor staging, Nx: unknown node involvement staging, Tumor staging was based on UICC, 7^th^ edition (2009).

### Treatment characteristics

#### Radiotherapy characteristics

Radiotherapy was delivered in pre-operative (n = 477, 80.5%), post-operative (n = 75, 12.6%) or exclusive (n = 41, 6.9%) setting. In patients undergoing a pre-operative radiotherapy, median rectal EQD2 was 49.2 Gy (Q1-Q3 = 44–50), with a median duration of 4.9 weeks. In post-operative and exclusive radiotherapy, median rectal EQD2 was 47.7 Gy (Q1-Q3 = 43.9–50) in both programs, with a median duration of 4.6 and 4.4 weeks, respectively. The median dose *per* fraction was 2 Gy for pre- and post- operative radiotherapy, and 2.5 Gy for exclusive radiotherapy. In pre-operative setting, median time from radiotherapy to surgery was 6.7 weeks (Q1-Q3: 6–7.5). For patients with post-operative radiotherapy, median time from surgery to radiotherapy was 10.3 weeks (Q1–Q3: 7.8–14.9). Radiotherapy characteristics are detailed in Table 2.

**Table 2:** Radiotherapy characteristics (n = 593 patients).

Pre-operative Radiotherapy	
Number of patients, n (%)	477 (80.5)
Median rectal EQD2, Gy (Q1-Q3)	49.2 (44–50)
Median duration, weeks (Q1-Q3)	4.9 (4.3–5.1)
Median dose per fraction, Gy/fr (Q1-Q3)	2 (1.8–2.1)
Median time to surgery, weeks (Q1-Q3)	6.7 (6–7.5)
Fractionation	
*Normofractionated schemes (1.8–2.4 Gy/fr), n (%)*	362 (61)
*Hypofractionated schemes (*≥*2.5 Gy/fr), n (%)*	115 (19.5)
**Post-operative Radiotherapy**	
Number of patients, n (%)	75 (12.6)
Median rectal EQD2, Gy (Q1-Q3)	47.7 (43.9–50)
Median duration, weeks (Q1-Q3)	4.6 (4.4–5.1)
Median dose per fraction, Gy/fr (Q1-Q3)	2 (1.8–2.4)
Median time to surgery, weeks (Q1-Q3)	10.3 (7.8–14.9)
Fractionation	
*Normofractionated schemes (1.8–2.4 Gy/fr, n (%)*	56 (9.4)
*Hypofractionated schemes (*≥*2.5 Gy/fr), n (%)*	19 (3.2)
**Exclusive radiotherapy**	
Number of patients, n (%)	41 (6.9)
Median rectal EQD2, Gy (Q1-Q3)	47.7 (43.9–50)
Median duration, weeks (Q1-Q3)	4.4 (2.9–5.3)
Median dose per fraction, Gy/fr (Q1-Q3)	2.5 (2–3)
Fractionation	
*Normofractionated schemes (1.8–2.4 Gy/fr,) n (%)*	18 (3)
*Hypofractionated schemes (*≥*2.5 Gy/fr), n (%)*	23 (3.9)

Q1: first interquartile, Q3: third interquartile, EQD2: 2Gy-per-fraction equivalent dose, n: number of patients, Gy/fr: Gray per fraction.

#### Chemotherapy characteristics

Chemotherapy was associated with radiotherapy in most of patients (n = 414, 69.8%), either in concurrent (n = 409, 69%) or sequential (n = 5, 0.8%) setting. Patients undergoing sequential chemo-radiation were either post-operative RC patients experiencing severe surgery complications or elderly post-operative patients. Concurrent chemotherapy was associated with 74.6% of pre-operative (n = 356/477), 52% of post-operative (n = 39/75) and 31.7% of exclusive (n = 13/41) radiotherapies. Oral or intravenous 5-FU was mainly used in concomitant setting (n = 331/409, 81%). Chemotherapy characteristics are reported in Table 3.

**Table 3:** Chemotherapy characteristics.

Whole set of patients, n (%)	593 (100)
Concurrent chemotherapy	
Number of patients, n (%)	409 (69)
Drug, n (%)	
*5FU (oral or intravenous)*	331 (55.8)
*FOLFOX*	60 (10)
*Xelox*	11 (1.9)
*Other*	7 (1.2)
Chemoradiotherapy setting, n (%)	
*Pre-operative*	356 (60)
*Post-operative*	40 (6.7)
*Exclusive*	13 (2.3)
Sequential Chemotherapy	
Number of patients, n (%)	5 (0.8)
Drug	
*FOLFOX*	3 (0.5)
*5FU (oral or intravenous)*	2 (0.3)
Chemoradiotherapy setting	
*Pre-operative*	0
*Post-operative*	5 (0.8)
*Exclusive*	0

Q1: first interquartile, Q3: third interquartile.

#### Acute and late toxicities

Due to acute toxicity, 4 patients (0.7%) did not complete the pre-planned radiotherapy program. One grade 5 acute toxicity was reported: the patient experienced an exclusive radiotherapy for a T4 tumor and had completed a myeloma chemotherapy one month before RC radiation initiation. His rectal tumor turned into abcess before radiotherapy, resulting in a fatal sepsis after 4 radiation courses. One grade 4 acute toxicity was reported, with an exclusive radiotherapy patient experiencing obstructive radiation proctitis. Acute infield toxicities were reported in 57.9% of pre-operative (262 grade 1–2, 14 grade 3), 53.3% of post-operative (35 grade 1–2, 5 grade 3), and 63.4% of exclusive (20 grade 1–2, 4 grade 3, 2 grade 4–5) radiotherapy patients. Acute grade 3 side-effects were gastro-intestinal (11 patients, with 10 receiving concomitant chemotherapy), cutaneous (7 patients, with 6 receiving concomitant chemotherapy) and urinary (1 patient receiving concomitant chemotherapy) toxicities.

No grade 5 late toxicities were reported. Two grade 4 infield late toxicities were reported, with ureter stenosis inducing chronic renal failure. The two patients experienced a pre-operative hypofractionated radiotherapy, with 2.5–2.7 Gy/fraction. One patient experienced a concurrent chemo radiation, with intravenous 5-FU. Late toxicities were reported in 13.8% of pre-operative (59 grade 1–2, 7 grade 3), 12% of post-operative (9 grade 1–2) and 2.4% of exclusive (one grade 2) radiotherapy settings. Late grade 3 toxicities were gastro-intestinal (6 patients experiencing fistula and stenosis, with 5 concurrent chemo radiation) and urinary (1 patient experiencing dysuria, who underwent concurrent chemo radiation).

### Efficacy

#### Global outcome

The median follow-up was 3.4 years. At the time of the analysis, 21.9% (n = 130) of the 593 included patients experienced a metastatic progression and 33% (n = 196) had died. In the subset of 552 patients undergoing rectal surgery, 29.2% (n = 161) had died at the end of follow-up. At the end of follow-up, 397 patients were alive (i.e. without a recorded date of death) in the whole set of patients, with 391 in the subset of patients who underwent a rectal surgery. In the whole set of patients, a total of 126 deaths were specifically caused by the RC. Median OS was 9.04 years (CI 95%: 7-NA). Median PFS was 6.7 years (CI95%: 4-NA). Out of the 552 patients undergoing a curative surgery, 54 had a pathological complete regression (9.8%).

#### Prognostic factors of OS in the whole set of patients: univariate and multivariate analysis

**Figure 1:**
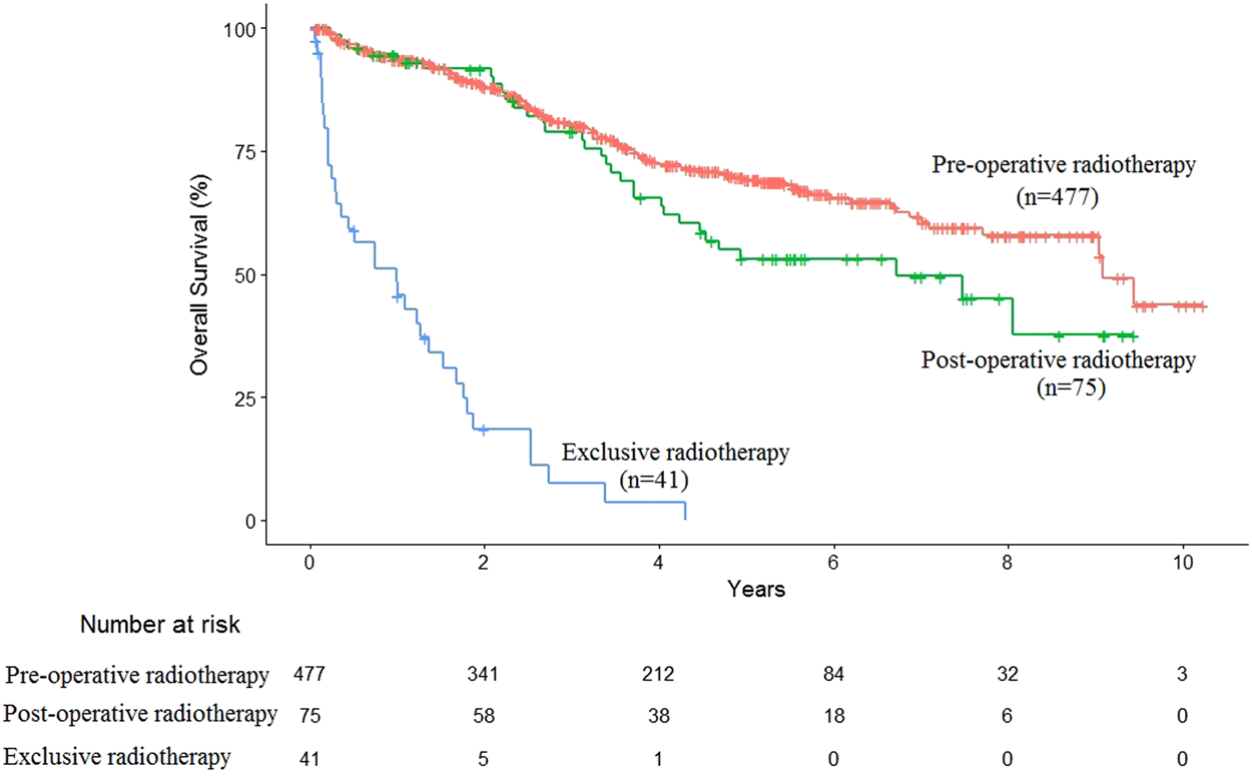
Overall survival of patients experiencing pre-operative, post-operative or exclusive radiotherapy.

As for radiotherapy characteristics, pre-operative or post-operative radiation setting was not correlated with significantly different outcome, with median OS of 9.1 years (CI95%: 9-NA) *vs*. 6.7 years (CI95%: 4.2-NA), p = 0.065, respectively (Fig. 1**)**. Exclusive radiotherapy was significantly associated with decreased survival with respect to pre-operative radiotherapy, with median OS of 1 year (CI95%: 0.36–1.7), p < 0.001 (Fig. 1). No rectal or pelvis radiation dose threshold was significantly correlated with OS in univariate analysis. The univariate analysis provided potential (p < 0.2) OS predictive factors, reported in Table 4. Although significant in univariate analysis, the complete tumor resection, the number of pathologically-proven involved lymph nodes, the vascular invasion and the exclusive radiotherapy could not be included in the multivariate analysis as they were correlated, by construction, with tumor resection. The impact of the selected factors on OS was thus studied using a Cox multivariate analysis **(**Table 4**)**. Regarding patients characteristics, age (>70 years old) was an independent risk factors of death (HR = 3.54 CI 95% (2.28–5.48), p < 0.001) and a correct nutritional condition (BMI > 18.5) was an independent protective factor of death (HR = 0.37 CI 95% (0.16–0.85), p = 0.02). Regarding tumor characteristics, stage III and poorly differentiated tumors were independent risk factors of death, with HR = 1.78 CI 95% (1.17–2.70), p = 0.007 and HR = 2.98 CI 95% (1.52–5.72), p = 0.001 respectively. Finally, tumor resection was the most important independent protective factor, with HR = 0.08 CI 95% (0.04–0.15), p < 0.001.

**Table 4:** Prognostic factors for overall survival in the whole set of patients (n = 593 patients).

Variable	Tested *vs*. Adverse criteria	UNIVARIATE ANALYSIS		MULTIVARIATE ANALYSIS	
		Hazard Ratio (95% CI)	p-value (log-rank test)	Hazard Ratio (95% CI)	p-value (cox model)
Age	>70 (n = 277) *vs*. ≤70 (n = 316)	3.44 (2.53–4.68)	**<0.001**	3.54 (2.28–5.48)	**<0.001**
Gender	Female (n = 231) *vs*. Male (n = 362)	1.04 (0.77–1.38)	0.812		
WHO performance status	2–3 (n = 44) *vs*. 0–1 (n = 549)	3.4 (2.27–5.09)	**<0.001**		
Body Mass Index	≥18.5 (n = 572) *vs*. < 18.5 (n = 21)	0.63 (0.31–1.30)	**0.20**	0.37 (0.16–0.85)	**0.02**
Tumor stage	Stage III (n = 315) *vs*. I-II (n = 241)	1.43 (1.05–1.94)	**0.022**	1.78 (1.17–2.70)	**0.007**
Rectal tumor location	Upper (n = 88) *vs*. Middle (n = 249)	1.32 (0.89–1.96)	**0.170**		
	Lower (n = 252) *vs*. Middle (n = 249)	1.18 (0.85–1.61)	0.312		
Tumor differentiation	Poor (n = 31) *vs*. Well/Moderate (n = 507)	1.82 (1.05–3.15)	**0.032**	2.98 (1.52–5.72)	**0.001**
Tumor resection	Yes (n = 552) *vs*. No (n = 41)	0.08 (0.05–0.12)	**<0.001**	0.08 (0.04–0.15)	**<0.001**
Complete tumor resection	Yes (n = 476) *vs*. No (50)	0.36 (0.24–0.54)	**<**0.001		
ypCR	Yes (n = 54) *vs*. No (n = 387)	0.57 (0.29–1.12)	0.104		
Number of pathologically-proven involved lymph nodes	N1 (n = 126) *vs*. N0 (n = 350)	2.19 (1.5–3.19)	**<**0.001		
	N2 (n = 63) *vs*. N0 (n = 350)	4 (2.61–6.14)	**<**0.001		
Vascular invasion	Yes (n = 137) *vs*. No (n = 352)	1.79 (1.27–2.52)	**<**0.001		
Perineural invasion	Yes (n = 17) *vs*. No (n = 104)	1.51 (0.69–3.31)	0.302		
Radiotherapy setting	Post-(n = 75) *vs*. Pre-operative (n = 477)	1.44 (0.97–2.12)	0.065		
	Exclusive (n = 41) *vs*. Pre-operative (n = 477)	13.30 (8.89–19.85)	**<**0.001		
Radiotherapy characteristics	Rectal EQD2 ≥ 45 Gy (n = 417) *vs*. < 45 Gy (n = 176)	0.78 (0.51–1.21)	0.267		
	Pelvis EQD2 ≥ 40 Gy (n = 536) *vs*. < 40 Gy (n = 57)	0.93 (0.68–1.29)	0.678		
	Hypofractionated (n = 157) *vs*. Normofractionated (n = 436)	1.51 (1.02–2.23)	**0.038**		
Concomitant chemotherapy	Yes (n = 409) *vs*. No (n = 184)	0.40 (0.30–0.52)	**<0.001**		

95% CI: 95% confidence interval; ypCR: Pathological complete response. EQD2: 2GY-per-fraction equivalent dose. All p-values ≤ 0.2 in univariate analysis were tested in multivariate analysis, except variables related to surgery since they substantially limited the number of patients and except correlated variables (correlation with p < 0.001). Finally, only bold typed values were tested in multivariate analysis.

The sum of sub-populations might be inferior to 593 in some cases because of missing data.

#### Prognostic factors of OS in patients undergoing tumor resection: univariate and multivariate analysis

The univariate analysis provided potential (p < 0·2) OS predictive factors. Although significant in univariate analysis, vascular invasion, incomplete tumor resection, concurrent chemotherapy, ypCR and performance status could not be included in multivariate analysis because of correlation with other variables. A poor tumor differentiation was an independent prognosis factor of shortened OS (HR = 3.43 CI 95% (1.65–7.15), p < 0.001). The degree of node involvement was also an independent risk factor of death, since N1 staging was associated with an HR = 2.11 CI 95% (1.39–3.22), p < 0.001 and N2 staging with HR = 3.04 CI 95% (1.86–4.97), p < 0.001. Finally, the most important independent predictive factor of death was the age with HR = 4.66 CI 95% (3.04–7.14), p < 0.001 for patients >70 years. Results of univariate and multivariate analyses are reported in Table 5.

**Table 5:** Prognostic factors for overall survival in patients undergoing tumor resection (n = 552 patients).

Variable	Tested *vs*. Adverse criteria	UNIVARIATE ANALYSIS		MULTIVARIATE ANALYSIS	
		Hazard Ratio (95% CI)	p-value (log-rank test)	Hazard Ratio (95% CI)	p-value (cox model)
Age	>70 (n = 243) *vs*. ≤70 (n = 309)	3.28 (2.35–4.58)	**<0.001**	4.66 (3.04–7.14)	**<0.001**
Gender	Female (n = 211) *vs*. Male (n = 341)	0.98 (0.71–1.34)	0.86		
WHO performance status	2–3 (n = 28) *vs*. 0–1 (524)	2.61 (1.53–4.45)	**<**0.001		
IMC	≥18.5 (n = 411) *vs*. < 18.5 (n = 19)	0.74 (0.33–1.69)	0.48		
Tumor stage	Stage III (n = 293) *vs*. I-II (n = 227)	1.48 (1.06–2.08)	**0.02**	1.38 (0.94–2.03)	0.10
Rectal tumor location	Upper (n = 83) *vs*. Middle (n = 236)	1.39 (0.90–2.14)	**0.13**		
	Lower (n = 229) *vs*. Middle (n = 236)	1.18 (0.83–1.67)	0.35		
Tumor differentiation	Poor (n = 27) *vs*. Well/Moderate (n = 475)	1.60 (0.84–3.05)	**0.15**	3.43 (1.65–7.15)	**<0.001**
Complete tumor resection	Yes (n = 476) *vs*. No (n = 50)	0.36 (0.24–0.54)	**<**0.001		
ypCR	Yes (n = 54) *vs*. No (n = 387)	0.57 (0.29–1.12)	0.10		
Number of pathologically-proven involved lymph nodes	N1 (n = 126) *vs*. N0 (n = 350)	2.19 (1.50–3.19)	**<0.001**	2.11 (1.39–3.22)	**<0.001**
	N2 (n = 63) *vs*. N0 (n = 350)	4 (2.61–6.14)	**<0.001**	3.04 (1.86–4.97)	**<0.001**
Vascular invasion	Yes (n = 137) *vs*. No (n = 352)	1.79 (1.27–2.52)	**<**0.001		
Perineural invasion	Yes (n = 17) *vs*. No (n = 104)	1.51 (0.69–3.31)	0.30		
Radiotherapy setting	Post-operative (n = 75) *vs*. Pre-operative (n = 477)	1.44 (0.98–2.12)	**0.07**		
Radiotherapy characteristics	Rectal EQD2 ≥ 45 Gy (n = 392) *vs*. < 45 Gy (n = 160)	0.99 (0.69–1.43)	0.97		
	Pelvis EQD2 ≥ 40 Gy (n = 505) *vs*. < 40 Gy (n = 47)	0.94 (0.56–1.57)	0.81		
	Hypofractionated (n = 133) *vs*. Normofractionated (n = 419)	1.08 (0.67–1.76)	0.75		
Concomitant chemotherapy	Yes (n = 396) *vs*. No (n = 156)	0.46 (0.34–0.63)	**<**0.001		

95% CI: 95% confidence interval; ypCR: Pathological complete response. EQD2: 2GY-per-fraction equivalent dose. All p-values ≤ 0.2 in univariate analysis were tested in multivariate analysis, except correlated variables (correlation with p < 0.001). Only bold typed values were tested in multivariate analysis. The sum of sub-populations might be inferior to 552 in some cases because of missing data.

## Discussion

The present article retrospectively identified predictive factors of OS in patients undergoing radiotherapy for a non-metastatic RC. As local radiotherapy is manly indicated in locally advanced tumours (mainly T3-T4 or N+) of lower and middle rectum, stage III tumours were over-represented in the present population. Moreover, over 80% of patients underwent pre-operative chemoradiation, which is very high compared to most institutions. Whereas post-operative chemoradiation is preferred in North America and in other countries, pre-operative chemoradiation is the standard of care in France, based on studies suggesting a better observance, tolerance and local efficacy of preoperative chemoradiation^2,3,11,12^. Furthermore, therapeutic strategies were systematically discussed in multidisciplinary teams (including at least a medical oncologist, a radiation oncologist and a surgeon) before treatment initiation.

Tumor resection was the most important predictive factor of survival in the whole set of patients, highlighting the key role of surgery in the management of the RC. Other previously described risk factors were identified with the age, the node involvement and the tumor differentiation^13,14^. However in literature, the impact of age on RC outcome is still debated since elderly patients seem less likely to undergo optimal anticancer treatment (tumor resection, pre-operative treatments) than younger patients^15^. In our series, age >70 years was correlated with a lower rate of concurrent chemotherapy and a poorer ECOG performance status, which can partly explain the poor outcome of elderly patients. Therefore, the exclusive hypofractionated accelerated radiotherapy (25 Gy/5fractions in 5 consecutive days) could probably be a major option for the geriatric population since it makes possible not to perform chemotherapy without prognosis impairment, and guarantees pre-operative treatment completion^16^. In our series, hypofractionated programs were widely used (30–50% of patients, depending on the radiotherapy setting, Table 2), without toxicity or overall survival impairment (Tables 4 and 5). However, to date, no radiotherapy program has been specifically validated in elderly patients. The PRODIGE-NACRE trial (NCT02551237) is currently prospectively investigating adapted to elderly radiotherapy programs, recruiting patients >75 years old with a stage II-III RC, randomizing them either in a pre-operative chemo-radiotherapy (50 Gy/25 fractions + capecitabine) or in a pre-operative hypofractionated radiotherapy (25 Gy/5 fractions). This trial will probably clearly define the best option for elderly patients in the next few years. Furthermore, in connection with frailties, an impaired nutritional condition (BMI ≤ 18.5) was an independent risk factor of death in the whole set of patients, proving the importance of developing personalized supportive care in RC. Taken together, these results suggest the absolute necessity to carry out an onco-geriatric evaluation before any treatment to define the best therapeutic sequence in elderly patients, including supportive care before any anticancer treatment.

Regarding radiotherapy characteristics, the present study highlighted heterogeneous prescription behaviours, with dozens of different programs in the 593 patients. However, only minor differences were noticed most of the time, explaining the coherent median rectal EQD2 with a thin inter-quartile range. This result probably explains why no dose threshold could be evidenced in the univariate/multivariate analyses, as the number of patients with rectal EQD2 ≤ 40 Gy was extremely scarce (n = 10, 1.6%). Besides, studies analyzing a dose escalation only proved that high doses (>40 Gy) increased complete pathological tumor response and local control but not overall survival^17,18^. The pre- or post-operative radiotherapy setting did not induced a statistically relevant difference regarding OS (p = 0.065) in the present study. However, the inferiority of post-operative radiotherapy was clearly shown on local control and toxicity in randomized controlled trials^2,3^, and should therefore be avoided as much as possible.

When considering only patients experiencing tumor resection, two pathologic independent predictive factors of poor OS were identified with poor tumor differentiation and node involvement. Although these two factors were previously described, the present analysis reveals their major importance in real-world patients. Although an adjuvant chemotherapy was performed in case of node involvement, patients still had an impaired OS. This step is critical in qualifying the bad-prognosis sub-population to treatment intensification, maybe with even more intense treatment than the current adjuvant chemotherapy. However, to date, the adjunction of cetuximab to pre-operative chemoradiation resulted in increased toxicity and insufficient efficacy (Trial ACCORD 14/0604). Other EGFR inhibitors (panitimumab, gefitinib) also resulted in disappointing results, possibly because of antagonist effects between 5-FU and EGFR therapies^19^. The identification of biomarkers of radioresistance, based on the association between tumor genetic profiling and patient local and distant outcomes might be a key to provide new therapeutic targets. This work will be carried out by our team, based on the cohort of the ProfiLER clinical trial (NCT 01774409).

Finally the present study featured, by definition, unavoidable biases. The retrospective nature is of course a major limitation. Furthermore, the short follow-up (<5 years) might have masked some late toxicities and might have biased the estimation of OS. The number of poorly differentiated tumours is low (5.2%) and may be related to the high number of missing data regarding the tumour differentiation (9.3%). These two elements might therefore have induced a bias in some results. The absence of information regarding the local control prevented us from identifying risk factor of local relapse. A few stage I patients underwent radiotherapy and were therefore included in this study since the decision was justified by multidisciplinary team.

## Conclusion

The present retrospective study identified independent predictive factor of overall survival in one of the largest cohort of real-world RC patients in literature. The age >70 years was the most important predictive factor of death, probably highlighting the previously reported sub-optimal management of care in elderly RC patients^15,20,21^. Poor tumor differentiation and node involvement were identified as major predictive factor of poor OS. The research in treatment intensification and in identification of radioresistance biomarkers should therefore probably be focused on this particular subset of patients. Finally, prescriptions of radiotherapy could probably be harmonised, regarding their current heterogeneousness. Although the retrospective nature of the study is a limitation, as well as the fact that the identified predictive factors for overall survival were not novel and finally quite predictable, this work reflect real life management of care and global outcomes of locally advanced rectal cancer patients.

## Electronic supplementary material

Supplementary Dataset 1
